# A Review on Solid Dispersion and Carriers Used Therein for Solubility Enhancement of Poorly Water Soluble Drugs

**DOI:** 10.34172/apb.2020.044

**Published:** 2020-05-11

**Authors:** Avinash Ramrao Tekade, Jyoti Narayan Yadav

**Affiliations:** Department of Pharmaceutics, Marathwada Mitra Mandal’s College of Pharmacy, Thergaon, Pune, Maharashtra- 411033, India.

**Keywords:** Hydrophillic carriers, Mucilage’s, Natural gums, Solid dispersions, Solubility, Solubility enhancement

## Abstract

A large number of hydrophilic and hydrophobic carriers in pharmaceutical excipients are available today which are used for formulation of solid dispersions. Depending on nature of carriers the immediate release solid dispersions and/or controlled release solid dispersions can be formulated. Initially crystalline carriers were used which are transformed into amorphous solid dispersions with enhanced properties. The carriers used previously were mostly synthetic one. Recent trend towards the use of natural carriers have replaced the use of synthetic carriers. This review is the overview of various synthetic, natural, semisynthetic, modified natural hydrophilic carriers used for formulation of solid dispersions.

## Introduction

Absorption of drug and its therapeutic effectiveness get affected by solubility which is a significant physicochemical factor. Poor aqueous solubility can leads to failure in formulation development process. The main reason behind inadequate bioavailability of drug is its low dissolution rate and low solubility in aqueous medium.^1^ A large number of hydrophilic carriers are explored today which have shown significant results for solubility enhancement. Nowadays, most of the drug substances were innovated but the venture to improve the solubility and dissolution of hydrophobic drug substances remain one of the trickiest tasks in drug development. Dissolution of drug in aqueous medium like gastric fluid is important to get better absorption and bioavailability for orally administered drug. Therefore, to progress bioavailability of poorly water soluble compounds like biopharmaceutical classification system class II and IV drugs, polymer matrix of various origin can be used. Various solubility enhancement methods have been introduced to triumph over this problem.^1^ There are several techniques for solubility enhancement which can be categorized into physical modification, chemical modifications for the drug substance, and other techniques^2^ which are listed in Table 1.

**Table 1:** Techniques for solubility enhancement of poorly water soluble drugs

**Techniques for solubility enhancement** ^2-5^	
Physical modification	A) Reduction Particle size
	a) Micronization
	b) Nanosuspension,
	B) Modification of the crystal habit
	C) Solid dispersions
	a) Eutectic mixtures
	b) Solid solutions
	c) Amorphous solid solutions
	d) Glass solutions and glass suspension
	e) Cryogenic techniques.
Chemical modification	A) Change of pH,
	B) Use of buffer,
	C) Derivatization,
	D) Complexation,
	E) Salt formation.
Miscellaneous methods	A) Supercritical fluid process,
	B) Use of adjuvant like surfactant, solubilizers, cosolvency, hydrotropy, and novel excipients.

One of the most promising and efficient techniques for solubility enhancement is solid dispersion formulation. According to Chiou and Riegelman, solid dispersion systems can be defined as ‘the dispersion of one or more active ingredients in an inert carrier or matrix at solid state prepared by the melting [fusion], solvent, or melting-solvent method’. The drug is hydrophobic in nature whereas matrix is hydrophilic. Solid dispersion can be classified as simple eutectic mixtures, solid solutions, glass solutions and glass suspensions, amorphous precipitation in a crystalline carrier, compound or complex formations.^3^ However, several modifications have been done in classification systems by various researchers which will be discussed in the following.

## Classification of solid dispersions

On the basis of recent advancement in solid dispersion, they can be classified as:

## First generation solid dispersion

The solid dispersions which could be prepared by using crystalline carriers are categorized as the first generation solid dispersions.^6^ Examples of used crystalline carriers are urea and sugars.^7^ In this type, thermodynamically stable crystalline solid dispersion get formed which releases the drug slowly.^6^ The dissolution rate is faster in case of amorphous solid dispersions (ASDs) as compared to crystalline sold dispersions. The first reported solid dispersion was eutectic mixture or monotectic mixture. In case of eutectic mixture melting point of dispersion is lower than the melting point of carrier and drug the melting point of drug and carrier are constant in case of monotectic mixture. In cooling process of eutectic mixture, the drug and carrier will crystallize simultaneously therefore it is preferred over the monotectic mixture.^8^ At the specific composition in eutectic mixture where drug crystallizes out is referred as eutectic point, and the mixture consists of fine crystals of two components.^6^ Small particle size will results in increased specific surface area which generally improves rate of dissolution and oral absorption of poorly water soluble drugs.^3^ Moreover, the number of studies having exact eutectic composition in solid dispersion is very limited.^8^

Based on the extent of miscibility between the two components or the crystalline structure of solid solution they are of two kinds. One is continuous [or isomorphous, complete, unlimited] solid solutions and the other discontinuous [or restricted, partial, limited, complete] solid solutions. They can also be classified into two groups- substitutional solid solutions in which the solute molecule substitutes the solvent molecule in the crystal lattice of the solid solvent. Whereas, in case of interstitial solid solutions, the solute molecule occupies the interstitial space of the solvent lattice.^3^ The disadvantage of first generation solid dispersion is forming crystalline solid dispersion as they were prepared using crystalline carriers like urea and sugars. Crystalline solid dispersions were more thermodynamically stable which lowers their dissolution rate as compared to amorphous one.^9^ Okonogi et al^10^ has studied the effect of urea and mannitol on crystallinity of ofloxacin. The higher solubility and dissolution rate were observed in case of urea based solid dispersions than mannitol based solid dispersions because urea reduced the crystallinity of ofloxacin more than mannitol proved by PXRD and DSC results.

## Second generation solid dispersion

These contain amorphous carriers like PVP, PEG, cellulose derivatives, etc.^7^ Second generation solid dispersions were found more effective than first generation solid dispersions (SD) because of their thermodynamic stability.^9^ According to the physical state of drug, ASDs can be classified as amorphous solid suspensions and amorphous solid solutions [glass solutions]. Amorphous solid suspensions consist of two separate phases while amorphous solid solutions contain molecularly homogenous mixture of both the drug and amorphous carriers. Amorphous carriers can be synthetic polymer or natural polymer.^8^ Amorphous solid suspensions can be formulated in case of drugs with limited carrier solubility or high melting point. In case of second generation solid dispersions because of forced solubilization of the drug in the carrier the drug is in its supersaturated state.^9^ Due to increase in chain length or molecular weights of polymers the aqueous solubility of polymers get decrease and viscosity get increased. Prevention of recrystallization of drugs in manufacturing, storage and dissolution process can be achieved by using high viscosity polymers. Moreover, the use of high viscosity polymer can delay the dissolution rate of drug in aqueous medium. The major problem regarding second generation solid dispersion is drug precipitation and recrystallization which affect the *in vitro* or *in vivo* drug release.^8^

## Third generation solid dispersion

The dissolution profile of drug can be improved using third generation solid dispersions which consists of carriers having surface activity or emulsifying properties.^9^ Use of special type of carrier for formulation of solid dispersions will overcome precipitation and recrystallization problems. Use of surfactant or emulsifiers not only improve the dissolution profile of drug but also improves physical and chemical stability of drug in solid dispersion. Examples of these carriers are inulin, Gelucire, poloxamer, etc.^8^ The physical and chemical stability of solid dispersion get enhanced by preventing nucleation and agglomeration.^9^ The selection of surface active agent or another polymer is based on dissolution or stability profile of drug, i.e. surfactant is used when faster dissolution is required while polymer with higher Tg may be used when prevention of re-crystallization is needed.^8,11^

## Fourth generation solid dispersion

These type of dispersions can be referred as controlled release solid dispersions (CRSD). It contain poorly water soluble drug with a short biological half life.^8^ The carrier used are either water soluble carrier or water insoluble carrier.^7^ Solubility enhancement and extended release of drug in controlled manner are the two targets in CRSD.^8^ The water soluble carriers used in CRSD are ethyl cellulose, Eudragit RS, Eudragit RL, HPC, etc.^7^

On the basis of physical state and molecular arrangement of active pharmaceutical ingredient (API) and carrier, binary solid dispersions can be divided into six distinct systems as follows:

Meng et al have classified solid dispersions into six groups Class C–C, Class C–A, Class A–C, Class A–A, Class M–C, Class M–A based on physical state and molecular arrangement of both API and carrier (Table 2). Further efforts are needed to shape a clear classification system and correlate it with the performance of solid dispersion in terms of solubility and stability.^12^

**Table 2:** Classification According to Physical State and Molecular Arrangement of API and Carrier

**Class**	**API**	**Carrier**
C-C	Crystalline	Crystalline
C-A	Crystalline	Amorphous
A-C	Amorphous	Crystalline
A-A	Amorphous	Amorphous
M-C	Molecularly dispersed	Crystalline
M-A	Molecularly dispersed	Amorphous

The aim of this article is to update on information regarding hydrophilic carriers used in solubility enhancement by using various solid dispersion methods. Here, hydrophilic carriers were classified on the basis of its origin like synthetic hydrophilic carrier, natural hydrophilic carriers, modified natural hydrophilic carriers and semi-synthetic hydrophilic carriers. Initially crystalline carriers like urea, sugars etc. were used in formulation of solid dispersions which have been changed to amorphous carriers including polymers. Therefore, mostly used form of solid dispersions is the ASDs. Use of various polymeric carriers affects dissolution characteristics of dispersed drug. Water soluble carrier results in a fast drug release while poorly soluble or insoluble carrier will retard the drug release from the matrix.^8^

## Mechanisms of incorporation of drug into polymer

Carriers used in solid dispersions are polymers. When drug and polymer are in intimate contact then drug occupy void spaces between polymeric chain and makes polymer chain relatively flexible. For example, in case of hot melt extrusion process, polymer is allowed to heat up to some extent that the heat given is responsible for loosening of polymer chain and incorporation of drug molecule into it. While in spray drying method the solvent used in process is responsible for weak cohesive inter and intra molecular interactions of polymer chain and resulting in formation of solvent polymer interactions. After this, drug molecules dissolved in solvent are incorporated into the loosened polymer chains.

Antiplasticization effect is observed when the mechanical properties of substance changes into stiff and brittle when another substance is added. In another way it can be explained as, compound with low Tg of resulting mixture would fall somewhere in between the Tg’s of both compounds. In this case drug undergoes antiplasticization. Where as polymer undergoes plasticization as its Tg decreases.^13^

## Drug release mechanism from solid dispersion

Dissolution performance of solid dispersion after its oral administration in the form of tablet, capsules, etc. will give proper idea about ultimate success. One of the successful approaches for enhancement of solubility of poorly soluble drug is conversion of crystalline form of drug to an amorphous from. For successful solid dispersion formulation major keys are supersaturation state maintenance and amorphous form stabilization. The problem regarding solid dispersions is precipitation of supersaturated drug which will ultimately affect its bioavailability.^13^ Increases stability and solubility of drug in medium is observed due to particle size reduction and reduced agglomeration.^14^ In supersaturating drug delivery system such as solid dispersion spring like effect is observed due to enhancement of dissolution rate of drug. At the stage of supersaturation decrease in dissolution rate is observed due to drug precipitation. Furthermore in such system parachute like effect is observed on dissolution profile of drugs when precipitation inhibitors are added.^12^ Drug controlled release and carrier controlled release are two types of mechanisms involved in drug release from immediate release solid dispersions. While in case of CRSD diffusion and erosion drug release mechanisms are observed depending on characteristics of polymer and the miscibility of the drug and carrier.^8^ If the carrier is soluble in the dissolution medium then the release of ASD is dissolution controlled mechanism while in case of insoluble carrier diffusion controlled mechanism is observed.^15^

## Carriers used in solid dispersion

Carriers plays major role in formulation of solid dispersion.^16^ They can be hydrophilic or hydrophobic or water swellable. Depending on their characteristics they can be used as release retardant or release enhancers.^8^ Also the dissolution characteristics of drug molecules are depend on nature of carriers.^17^ The criteria for selection of carrier are as follows:

- It should be water soluble or swellable, soluble in variety of solvents.
- It should be economical, pharmacologically inert, non-toxic.
- It should be heat stable.
- Chemically compatible with drug.

The better chemical stability of solid dispersion was observed in case of solid dispersions which are formulated using solvent based methods as compared to fusion based methods. Solvent based methods include spray drying, co-precipitation, etc. While hot melt extrusion, KinetiSol® dispersing technology, etc. comes under fusion based methods.^18^ Hydrophilic carriers used in solid dispersion are classified on the basis of their origin. Table 3 shows list of synthetic hydrophilic carriers.

**Table 3:** Synthetic Hydrophilic Carriers

**Name of hydrophilic carrier**	**Method used for preparation of SDs**	**Drug used**	**Conclusion**
Brij35 and Pluronic F-127 ^19^	Solvent evaporation method	Progesterone	Progesterone incorporation into Pluronic F-127 and Brij 35 micelles improved its aqueous solubility by ~20 folds
Carboxymethylcellulose and sodium alginate (ALG)^20^	Solvent evaporation method	Praziquantel	Improved solubility
Compritol 888 ATO^21^	Solvent evaporation method, Sustained release solid dispersions	Metformin HCL	Solid dispersion(SD) for controlled release of metformin were developed using compritol
Dextrin^22^	Spray-drying technique	Micronized amlodipine	Amlo-SD with and without SLS provided 2.8- and 2.0-fold increase in AUC respectively
Eudragit E, Plasdone S and Soluplus^23^	Hot melt extrusion (HME), freeze-drying (FD), and supercritical fluid (SF)	Theobromine	Improved dissolution profile
Eudragit S100^24^	Solvent evaporation method	Berberine HCL	Improved efficacy
Gelucire and Sorbitol^25^	Solvent method, Melt method	Ritonavir	Release of ritonavir from solid dispersion is more than that of pure drug
HPMC and PVA-PEG grafted copolymer^26^	Solvent evaporation method	*Kaempferia parviflora*	Solid dispersion technique can be applied for the production of herbal extracts
Mannitol^27^	Spray drying method	Diazepam	Improved dissolution behaviour of drug
Palm stearin based polyesteramide (PSPEA)^28^	Hot melt method	Mefenamic acid	Improved the release and solubility
Pectin: poly (vinyl pyrrolidone)^29^	Spray drying technique	Curcumin	The in vitro dissolution data showed many fold increase in dissolution rate
Poloxamer 188^30^	Hot melt method	Losartan potassium	A dissolution rate much higher than that of pure drug
Poloxamer 188, Poloxamer 407^31^	Kneading method, solvent evaporation method	Boswellic acid	Improved solubility of poorly water soluble boswellic acid
Poloxamer + d-α-tocopherol polyethylene glycol 1000 succinate (TPGS)^32^	Solvent evaporation method	Febuxostat	Solid dispersion exhibited a drug release rate that was 1.94 times greater than plain drug
Poly(2-ethyl-2-oxazoline)^33^	Solvent evaporation method	Glipizide	Glipizide solubility improved by 2.5 times with poly[2-ethyl-2-oxazoline] compared to about 1.8 times with pure drug
Poly(ethylene oxide) (PEO) (3400, 10000, 20000)^34^	Solvent evaporation method	Griseofulvin	Increase the dissolution rate and the bioavailability of poorly water-soluble drug
Polyethylene glycol (PEG) 4000^35^	Fusion method	Gliclazide	Dissolution rate improved 90% compared to pure drug
Polyethylene glycol (PEG) 4000^36^	Microwave induced solid dispersion	Glipizide	The solid dispersion matrix tablet displayed retarded drug release up to 99.320% in 12 h.
Polyethylene glycol 6000^37^	Microwave induced fusion method	Atorvastatin calcium	An increase in the solubility of atorvastatin was observed with increasing concentration of PEG 6000
Polyethylene glycol 8000 and polyethylene glycol 10000^38^	Solvent evaporation method	Flurbiprofen	Significant increase in drug release
Polyoxyethylene 40 stearate^39^	Solvent melt method	Cyclosporine A	Improved dissolution and bioavailability
Polyvinylpolypyrrolidone (PVPP)^40^	Solvent evaporation method	Oleanolic acid	The optimized SD's system could improve about 15 times of dissolution rate than that of free OLA in 5 min.
Polyvinylpyrrolidone (PVP)^41^	Melt quenching	Celecoxib	Improved *in vitro* and in vivo performance
Polyvinylpyrrolidone (PVP) K30: isomalt (Galen IQ 810)^42^	Spray drying method	Celecoxib	Increased dissolution rate and saturation solubility of celecoxib
PVP K-12, PVP K-17, PVP K-30, Copovidone^43^	Solvent evaporation method, Spray drying method	Chlortetracycline hydrochloride	The effect of preparation methods, polymer types and polymer concentrations were evaluated
PVP-VA64 + POLOXAMER 407^44^	Spray drying method	Rebamipide	Bioavailability and efficacy of rebamipide were increased significantly by solubility enhancement of the drug.
Sodium acetate^45^	Freeze drying	Docetaxel	Enhanced dissolution rate resulting in enhanced bioavailability
Soluplus^46^	Hot Melt Extrusion, Spray Drying	Telmisartan	Results demonstrated an improvement in solubility of TEL by 99 times as compared to pure drug in buffer pH 7.4
Soluplus^47^	Solvent evaporation technique	Edaravone	Self-nanomicellizing solid dispersion (SNMSD) of edaravone give 17.53 fold improve in aqueous solubility.
Tocopheryl PEG 1000-succinate (TPGS)^48^	Solvent evaporation method	Dutasteride	Dutasteride solid dispersion formulations improved the dissolution and oral bioavailability of drug because of hydrogen interactions between carrier and drug
Vinylpyrrolidone-vinyl acetate (VA 64), PVP, HPMC^49^	Solvent evaporation method	Dronedarone HCL	Improved dissolution rate and intestinal absorption
Vinylpyrrolidone-Vinyl Acetate Copolymer-64+ Sucrose laurate^50^	Surface modified SD's prepared by Spray drying method	Oxcarbazepine	Enhanced solubility
Urea^51^	Fusion method	Rofecoxib	The mean dissolution time (MDT) of rofecoxib decreased which results in enhanced dissolution rate.

In recent years, polymers those are derived from plant origin are getting tremendous interest because of their diverse pharmaceutical applications and also easy availability, biocompatibility, non-toxic nature, chemically inertness they are preferred over the synthetic ones. Demand for these substances is increasing and new sources are being developed. Polysaccharide, one of the most abundant industrial raw materials and have been the subject of intensive research due to their sustainability, bio-safety and bio-degradability. The natural gums are metabolic by-products of plants obtained from various parts of plant like seed, fruit, incised trunk (gummy exudate), etc.^52^ Various natural hydrophilic carriers used till date are given in Table 4.

**Table 4:** Natural hydrophilic carriers

**Name of hydrophilic carrier**	**Method used for preparation of solid dispersions**	**Drug used**	**Conclusion**
*Aegle marmelos* Gum^53^	Microwave induced fusion method, lyophilisation Technique.	Atorvastatin calcium	The SD's prepared using the lyophilization method displays faster dissolution rates compared with those prepared using other method
Alginate^54^	Solvent evaporation method	Lovastatin and Indomethacin	Significant improvement in drug dissolution and stability
Arginine^55^	Freeze drying	Simvastatin	Simvastatin-arginine (SMV-ARG) complex exhibited solubility enhancement by 12 000 fold in both acidic and alkaline dissolution media
Caffeine^56^	Solubility method	Celecoxib	Enhancement of solubility and dissolution rate was observed
Chitosan^57^	Solvent evaporation method	Abietic acid	Improved activity
Low molecular weight chitosan^58^	Solvent evaporation method	Tanshinone llA	Dissolution of Tanshinone llA increased about 368.2% compared with the pure drug
*Elaeagnus angustifolia* fruit powder, crospovidone, microcrystalline cellulose^59^	Cogrinding method	Piroxicam	Increased dissolution rate was observed
Skimmed milk^60^	Lyophilization	Simvastatin	In-vitro drug release studies exhibited a cumulative release of 86.69% as compared to 25.19% for the pure drug
Soybean seeds^61^	NA	Pioglitazone HCl	Increase in drug solubility
*Daucus carota* ^62^	Solvent evaporation and kneading method	Ambrisentan	Solid dispersion by kneading method showed better results than the solvent evaporation method.
Water soluble gelatin and egg albumin^63^	Kneading method	Nifedipine	Improved the wettability of the drug, and consequently enhanced dissolution rate
Gelatin 50PS^64^	Freeze drying	12 Active pharmaceutical ingredients	Gelatin 50PS was screened for its feasibility as carrier in the formulation of solid dispersions by lyophilization
Neem gum^65^	Solvent evaporation and kneading method	Atorvastatin	Enhanced dissolution rate and bioavailability
Oleaster powder, microcrystalline cellulose and crospovidone^66^	Cogrinding method	Ibuprofen	Oleaster powder is suggested as a potential hydrophilic carrier for increasing the drug release
Sericin^67^	Spray drying, solvent evaporation, ball milling	Lornoxicam, meloxicam and felodipine	Spray drying as an efficient technique for improvement in solubility [8 to 10 fold] and dissolution of drugs
Sodium alginate^68^	Grinding method	Telmisartan	The highest release rates were achieved by SD with higher proportions of sodium alginate about 19.5 fold increase in dissolution profile were observed.
Tamarind seed polysaccharide^69^	Co-grinding method, kneading method, solvent deposition method	Celecoxib	Enhanced dissolution rate
*Vigna radiata* extract^70^	Solvent evaporation method	Clopidogrel bisulfate	The maximum drug release was found in formulation was 96.12%

Natural gum polysaccharides are promising biodegradable, biocompatible materials for use in drug delivery systems. However, these materials have certain limitations, like uncontrolled rate of hydration, change in viscosity during shelf life, microbial contamination. Therefore to overcome this problems some modification have done. Modifications can be done in terms of physical modifications and chemical modifications. Physical methods for modification of involves use of dry heat, microwave technology, UV and gamma radiations. While chemical modifications involves carboxymethylation/carbomoylethylation in which free –OH groups replacement were done which leads to improved aqueous drug solubility.^71^ Generally natural carriers on modification using heating method will leads to change in its physical characteristics like viscosity, density, swelling index, water holding capacity, flow properties etc. Due to changes in these characteristics the improved results were obtained in case of modified natural carriers.^4^ Carboxymethylation of natural carriers increases their hydrophilicity and makes them more soluble in aqueous systems.^71^ Various modified hydrophilic carriers and semi synthetic carriers used are shown in Table 5 and Table 6.

**Table 5:** Modified natural hydrophilic carriers

**Name of hydrophilic carrier**	**Method used for preparation of SDs**	**Drug used**	**Conclusion**
Locust bean gum, Modified LBG (LBG/ MLBG)^72^	Kneading method, spray drying method, solvent wetting method, modified solvent evaporation method	Lovastatin	Increase in apparent solubility and increase in dissolution rate
Modified gum Karaya^73^	Solvent evaporation method	Glimepiride	Enhanced dissolution rate
Modified gum Karaya^74^	Co-grinding mixture, kneading mixture, solvent evaporation method	Nimodipine	Improvement of dissolution rate
Locust bean gum (LBG), modified LBG, Guar gum, Modified guar gum^75^	Solvent evaporation method	Glibenclamide	Modified forms of natural carriers could be potential carriers in dissolution rate enhancement of poorly soluble drugs
Guar gum, Modified guar gum^76^	Cogrinding mixtures	Licofelone	Enhanced dissolution rate and decreased particle agglomeration
Guar gum, Modified guar gum^77^	Microwave induced solid dispersions	Simvastatin	Optimized formulation showed 99.35±3.3.05% drug release, which is higher than that of MKT [97.77±2.51%]
Hupu gum, modified hupu gum^78^	Gel entrapment technique	Clopidogrel Bisulphate	Enhancement in solubility of Clopidogrel bisulphate
Hupu gum, modified hupu gum^79^	Cogrinding method	Pioglitazone HCL	Enhanced solubility & bioavailability
Modified fenugreek gum^80^	Co solvent precipitation method	Simvastatin	Used as solubility enhancer and stabilizer in solid dispersion preparation
Modified xanthan gum^81^	Kneading method	Pioglitazone hydrochloride	Cumulative drug release in optimized formulations of xanthan gum and modified xanthan gum was 85.37% and 99.21% respectively

**Table 6:** Semi-synthetic hydrophilic carriers

**Name of hydrophilic carrier**	**Method used for Preparation of SD's**	**Drug used**	**Conclusion**
Chito-oligosaccharide^82^	Spray drying method.	Hesperidin	Enhanced solubility and antioxidant activity
Ethyl cellulose, hydroxypropyl methylcellulose^83^	Solvent evaporation method	Hydrochlorothiazide	Improved dissolution rate
Hydroxypropyl methylcellulose (HPMC E5 LV)^84^	Spray drying method	Irbesartan	Enhancing solubility and dissolution rate
Hydroxypropyl methylcellulose (HPMC E5 LV)^85^	Microwave induced fusion method	Raloxifene HCL	Increased solubility as well as in vitro drug dissolution
HPMC: MESOPOROUS SILICA (ternary ASD)^86^	Hot melt extrusion	Indomethacin	Enhanced dissolution rate and physical stability

## Advantages of solid dispersions

**Figure 1:**
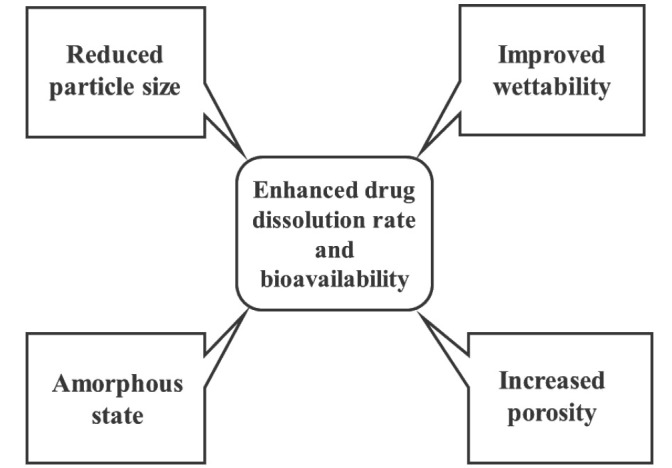
Advantages of solid dispersions.

The main objective behind formulation of solid dispersions is to enhance solubility of drug and thereby enhancement of its *in vitro* dissolution rate and bioavailability as well as developing controlled release solid dispersions.^87^ The factors affecting drug solubility are its particle size, porosity, wettability, etc.^9^ Various advantageous properties of solid dispersions are showed in Figure 1.

## Characterization of solid dispersions

Solid dispersions are mainly known for their use in dissolution rate and bioavailability enhancement. The enhanced dissolution rate can be examined by using standard dissolution methods which involves use of USP dissolution test apparatus. Another parameter studied in case of solid dispersion is to detect the physical state of drug and polymer, like state of material (amorphous or crystalline) and degree of crystallinity.^8^ Many analytical and instrumental technique are used to characterize solid dispersions. Techniques used for characterization are can be thermal methods, spectroscopic methods, microscopic methods, microthermal analysis, macroscopic techniques, etc.^88^ Their significance and characteristic features are listed in Table 7.

**Table 7:** Various methods for characterization of solid dispersions and its significance

**Characteristics**	**Methods used**	**Significance**
Physical state examination	Differential scanning calorimetry, Powder X-ray diffraction, Hot stage microscopy, Humidity stage microscopy	To find out physical state of sample, crystallinity and degree of crystallinity of drug, polymer, solid dispersion.
Surface microscopy	Scanning electron microscopy, hot stage microscopy, polarized light optical microscopy	To examine microscopy and crystallinity
Structure elucidation	Solid state nuclear magnetic resonance spectroscopy, Fourier transform infrared spectroscopy	To investigate bonding between drug and carrier e.g. hydrogen bonding
Drug carrier interactions	Differential scanning calorimetry, Nuclear magnetic resonance spectroscopy, Fourier transform infrared spectroscopy	To study physical and chemical interactions between drug and polymer
Dissolution rate	Dissolution studies, dynamic solubility studies	To study rate and extent of drug release
Stability	Differential scanning calorimetry, nuclear magnetic resonance spectroscopy, Fourier transform infrared spectroscopy	To study physical and chemical interactions between drug and polymer during its manufacturing and storage period

## Conclusion

New chemical entities are mostly the poorly water soluble drugs. To overcome poor solubility problem, solid dispersions can be prepared using hydrophilic carriers. These carriers can be of synthetic or of natural origin. Major problem regarding solid dispersions is its stability which can also be overcome by using newly coming carriers and use of optimized manufacturing techniques. Industrial and academic research work have solving the scalability problem of solid dispersions. There are still several carriers which are not investigated so far. Therefore, studies on such carrier materials should be done for solubility enhancement.

## Ethical Issues

Not applicable.

## Conflict of interests

There is no conflict of interest.

## Acknowledgments

Authors are thankful to the Management and Principal of Marathwada Mitra Mandal’s College of Pharmacy, Thergaon, Pune, for motivation and infrastructural facilities for completing this review.
